# Treatment failure in adolescents and young people living with HIV: a cohort study

**DOI:** 10.1590/0034-7167-2025-0340

**Published:** 2026-06-22

**Authors:** Camila Moraes Garollo Piran, Alana Vitória Escritori Cargnin, Mariana Martire Mori, Marcelo da Silva, Sonia Silva Marcon, Ieda Harumi Higarashi, João Manuel Graça Frade, Marcela Demitto Furtado

**Affiliations:** I Universidade Estadual de Maringá. Maringá, Paraná, Brazil; II Instituto Politécnico de Leiria. Leiria, Portugal

**Keywords:** HIV, Human Immunodeficiency Syndrome, Lost to Follow-Up, Adolescent, Young Adult., VIH, Síndrome de Inmunodeficiencia Humana, Perdida de Seguimiento, Adolescente, Adulto Joven.

## Abstract

**Objectives:**

to analyze factors associated with poor adherence and loss to follow-up among adolescents and young people living with HIV.

**Methods:**

ambidirectional cohort study of 512 adolescents and young people with HIV receiving treatment at a specialized service in northwestern Paraná, Brazil. Poisson regression with robust variance was performed.

**Results:**

having a sexual partner, time between diagnosis and antiretroviral therapy initiation, and missed appointments were negatively associated with poor adherence. Age group of 20-24 years was positively associated with adherence. Drug use, an antiretroviral therapy regimen consisting of lamivudine+tenofovir+efavirenz, last unsuppressed viral load result before the outcome, time between diagnosis and antiretroviral therapy, and missed appointments were associated with loss to follow-up. Alcohol use, the first unsuppressed viral load test, and the last undetectable viral load test were positively associated.

**Conclusions:**

the various determinants involved in illness and treatment among adolescents and young people living with HIV were reiterated.

## RESUMEN

**Objetivos:**

analizar factores asociados a baja adherencia al tratamiento y pérdida de seguimiento en adolescentes y jóvenes que viven con el VIH.

**Métodos:**

cohorte ambispectiva de 512 adolescentes y jóvenes con VIH en tratamiento en servicio especializado del noroeste de Paraná, Brasil. Regresión de Poisson con varianza robusta.

**Resultados:**

tener pareja sexual, tiempo transcurrido entre el diagnóstico y inicio del tratamiento antirretroviral, y inasistencia a las citas se asociaron negativamente con una baja adherencia. El grupo de edad de 20 a 24 años se asoció positivamente. Consumo de drogas, régimen de tratamiento antirretroviral compuesto por lamivudina+tenofovir+efavirenz, carga viral no suprimida antes del desenlace, tiempo transcurrido entre el diagnóstico y inicio del tratamiento antirretroviral, e inasistencia a las citas se asociaron con la pérdida de seguimiento. Consumo de alcohol, una primera prueba de carga viral no suprimida y una última prueba de carga viral indetectable mostraron una asociación positiva.

**Conclusiones:**

se reiteraron los diversos factores determinantes interrelacionados con la enfermedad y tratamiento en adolescentes y jóvenes que viven con el VIH.

## INTRODUCTION

Human immunodeficiency virus (HIV) remains a challenging infection that significantly impacts healthcare systems, particularly due to the progression of HIV to Acquired Immunodeficiency Syndrome (AIDS) and its associated morbidity and mortality^(1)^.

Despite the benefits of antiretroviral therapy (ART), the Joint United Nations Programme on HIV/AIDS 2023 global report revealed that, of the 39.9 million people living with HIV (PLHIV) worldwide, only 77% are using ART^(2)^. Globally, the incidence of HIV among adolescents and young people has been 22.7 per 100,000 inhabitants (95% Confidence Interval (95%CI): 3.1 to -2.0)^(3)^. It is estimated that, of seven new HIV infections, two occur among adolescents and young people aged 15 to 24^(4)^.

Adolescents and young PLHIV frequently experience adverse treatment outcomes, such as poor adherence and loss to follow-up, in different settings^(5-7)^. Considering the magnitude of the problem, there is a global effort to end the HIV epidemic by 2030, which is even one of the targets of the United Nations Sustainable Development Goals^(8)^.

However, in the case of HIV infection, as with chronic diseases, individuals play a fundamental role in caring for their own health, and therefore must take responsibility for their self-care. In the context of HIV, treatment adherence is essential for successful outcomes, and this, in turn, is influenced by patients’ history, motivations, and commitment to their own quality of life^(9)^.

However, managing treatment among adolescents and young people is challenging, especially due to factors such as immaturity, therapeutic regimen, access to healthcare services, low socioeconomic status accentuated by the disease, and social stigma^(10,11)^.

A scoping review conducted by the authors identified that several intrinsic and extrinsic factors interfere with health monitoring of adolescents and young PLHIV; however, this remains insufficient. Despite the variety of studies, the need arose to address some gaps, using an ambispective cohort as a study method and coordinating data focused on the social determinants of health, which may be related to treatment failure.

## OBJECTIVES

To analyze factors associated with poor adherence and loss to follow-up among adolescents and young people living with HIV

## METHODS

### Ethical aspects

The study was developed in accordance with the guidelines established by Resolutions 466/12 and 510/16 of the Brazilian National Health Council, and was approved by the Research Ethics Committee. Records containing participant identification were replaced with codes and numbers to guarantee anonymity and preserve their identity.

### Study design, period, and location

This is an epidemiological, open-label, ambispective cohort study of adolescents and young adults living with HIV. The STrengthening the Reporting of OBservational studies in Epidemiology guidelines were used to guide the study in accordance with the EQUATOR network recommendations.

The study was conducted at the STI/HIV/AIDS and viral hepatitis outpatient clinic located in the northwest region of the state of Paraná, Brazil. This outpatient clinic offers three distinct services: a Testing and Counseling Center; a Specialized Care Service; and a Medication Dispensing Unit.

### Population or sample; inclusion and exclusion criteria

The sample was non-probabilistic (consecutive), composed of all adolescents and young people with International Classification of Diseases - indicator of statistics of mortality and morbidity - B20.0 to B24 referring to HIV/AIDS.

The eligibility criterion adopted was being adolescents and young people aged between 10 and 24 years old who sought care at the referral clinic for clinical and medication follow-up between January 1, 2017, and December 31, 2023. Only cases of sexual transmission were included. The year 2017 was chosen because, on June 7, 2016, in the United Nations General Assembly Political Declaration on Ending AIDS, countries committed to accelerating the response to fight HIV and end the AIDS epidemic by 2030^(12)^. The inclusion of participants until December 31, 2023, in turn, is justified by the need to follow the HIV/AIDS case for a period of time until outcome or up to 24 years, 11 months, and 29 days, sufficient time to investigate adherence, poor adherence, or loss to follow-up. Twenty-one participants were excluded from the study, including 19 young people who were diagnosed at age 24 but enrolled in the service at age 25, in addition to two cases due to deaths.

### Study protocol

Data were collected between June 2024 and January 2025 by reviewing the medical records of patients treated at the STI/HIV/AIDS and viral hepatitis outpatient clinic. The lead researcher, along with the research team (one doctoral student and one master’s student), scheduled appointments in advance with the clinic to avoid interfering with the department’s routine. A structured instrument was used to obtain the study variables. The instrument was developed according to information from Ministry of Health protocols, and underwent a simple assessment process by eight experts before data collection began.

The dependent variables considered for the study were poor adherence and loss to follow-up. In the case of HIV treatment, adherence is defined as attending scheduled appointments, undergoing requested follow-up tests, and picking up medications on the scheduled date. Therefore, poor adherence is defined as failure to pick up ART within seven days of the scheduled date. Abandonment/loss to follow-up refers to cases of failure to pick up antiretroviral medications from the pharmacy three months after the scheduled date and/or failure to return for appointments within six months^(13)^. Independent variables were grouped into thematic areas: sociodemographic; behavioral; clinical, immunological, and laboratory.

### Analysis of results, and statistics

Incidence rates for adherence, poor adherence, and loss to follow-up were estimated, stratified by individual and treatment variables. Calculation was performed using the proportion of observations for each subvariable in that outcome relative to the total number of observations for that characteristic, and the result was expressed as 100. These rates were accompanied by 95%CIs. Descriptive analysis also included the mean time (MT) of follow-up for each outcome, with the respective standard deviations (SDs).

Subsequently, Poisson regression models with robust variance were constructed to assess the association of study variables with “poor adherence” and “loss to follow-up” outcomes, using “adherence” as the reference category for comparison. Bivariate analysis considered variables with a p-value <0.25 in the Wald chi-square test for subsequent inclusion in the multiple models. Variables that were marginally significant in the bivariate analysis were assessed for multicollinearity before the next stage.

The construction of multiple models for each outcome considered the stepwise backward selection method, in which the variables selected in bivariate analysis were entered together and then removed one by one, depending on statistical significance. The final model for each outcome (“poor adherence” and “loss to follow-up”) was adjusted for the remaining variables (p<0.05), as well as for the “distance to service” variable, as it was understood that travel to the outpatient clinic could influence follow-up.

Relative risks (RR), accompanied by their 95%CIs, were used as measures of association between independent and dependent variables, allowing for the assessment of their magnitude and direction. It is important to note that variables with missing information were included in descriptive analysis but were not included in the regression models, as they could compromise the analysis integrity and reliability, preventing appropriate interpretation. All analyses were performed using the Statistical Package for the Social Sciences^®^ version 21.0.

## RESULTS

During the study period, 512 adolescents and young PLHIV met the study eligibility criteria, with 155 classified as adherent, 160 as poor adherent, and 197 as lost to follow-up. The median follow-up time for adolescent patients with adherence was 807.6 days (SD=617.4), while for those with poor adherence, it was 779.6 days (SD=785.5), and for loss to follow-up, it was 755.1 days (SD=595.8). Concerning young people, the MT for adherence was 538.4 days (SD=431.5), for poor adherence, 454.7 days (SD=396.0), and for loss to follow-up, 501.8 days (SD=458.4).

In relation to participants’ main characteristics, in the adherence group, the highest incidence rate was among males (32%), young people aged between 20 and 24 years (31.7%), white race/color (32.8%) and with more than 12 years of education (38.3%). Regarding the poor adherence group, there was a predominance of males (31.8%), age group between 15 and 19 years (40.6%), and non-white race/color (32.8%). In the loss to follow-up group, females (57.1%), age group from 20 to 24 years (38.6%), non-white race/color (44.015), and education <12 years (43%) predominated. Table 1 shows other characteristics of adolescents and young PLHI.

**Table 1:** Incidence rates of adherence, poor adherence, and loss to follow-up among adolescents and young people living with HIV, Maringá, Paraná, Brazil, 2025

Variable	Adherence		Poor adherence		Loss to follow-up	
	IR	95%CI	IR	95%CI	IR	95%CI
Sex						
Female	16.1	6.5; 25.7	26.8	15.2; 38.4	57.1	44.2; 70.1
Male	32.0	27.7; 36.3	31.8	27.5; 36.1	36.2	31.8; 40.6
Age group						
15-19 years	21.6	12.2; 31.0	40.6	29.4; 51.7	37.8	26.8; 48.9
20-24 years	31.7	27.4; 36.1	29.7	25.4; 34.0	38.6	34.0; 43.1
Marital status						
Without a partner	31.3	27.0; 35.6	32.0	27.7; 36.3	36.7	32.2; 41.1
With a partner	22.6	12.2; 33.0	25.8	14.9; 36.7	51.6	39.2; 64.1
Race/color						
White	32.8	28.1; 37.5	30.7	26.0; 35.3	36.5	31.7; 41.4
Non-white	23.1	16.0; 30.3	32.8	24.9; 40.8	44.0	35.6; 52.4
Education						
≤12 years	25.3	20.5; 30.1	31.6	26.5; 36.8	43.0	37.6; 48.5
>12 years	38.3	31.5; 45.1	30.6	24.2; 37.1	31.1	24.6; 37.6
Religion						
No	33.2	27.7; 38.7	32.5	27.1; 38.0	34.3	28.7; 39.8
Yes	26.6	20.9; 32.4	29.7	23.8; 35.6	43.7	37.2; 50.1
Sexual orientation						
Heterosexual	18.8	10.2; 27.3	21.3	12.3; 30.2	60.0	49.3; 70.7
Homosexual	32.9	28.2; 37.6	31.6	27.0; 36.2	35.5	30.7; 40.3
Bisexual	28.3	15.2; 41.3	45.7	31.3; 60.0	26.1	13.4; 38.8
Occupation						
Employed/self-employed	31.3	27.0; 35.7	30.9	26.5; 35.2	37.8	33.2; 42.3
Student/houseperson	26.7	15.5; 37.9	40.0	27.6; 52.4	33.3	21.4; 45.3
Unemployed	16.7	-0.6; 33.9	11.1	-3.4; 25.6	72.2	51.5; 92.9
Distance to service						
<5 km	29.9	22.3; 37.6	38.7	30.5; 46.8	31.4	23.6; 39.2
5-10 km	34.4	28.6; 40.1	28.6	23.2; 34.1	37.0	31.2; 42.9
>10 km	21.4	13.8; 29.0	28.6	20.2; 36.9	50.0	40.7; 59.3
No information	0.0	0.0; 0.0	0.0	0.0; 0.0	100.0	100.0; 100.0
Homeless person						
No	30.2	26.2; 34.2	31.6	27.5; 35.6	38.3	34.0; 42.5
Yes	40.0	-2.9; 82.9	0.0	0.0; 0.0	60.0	17.1; 102.9
Incarcerated person						
No	30.4	26.4; 34.4	31.4	27.3; 35.4	38.3	34.0; 42.5
Yes	20.0	-15.1; 55.1	20.0	-15.1; 55.1	60.0	17.1; 102.9
Alcohol use						
No	27.8	21.7; 33.8	31.1	24.8; 37.4	41.1	34.5; 47.8
Yes	32.0	26.8; 37.3	31.4	26.1; 36.6	36.6	31.2; 42.1
Tobacco use						
No	31.7	26.8; 36.6	33.7	28.7; 38.7	34.6	29.6; 39.6
Yes	27.4	20.6; 34.1	26.2	19.5; 32.8	46.4	38.9; 54.0
Drug use						
No	31.6	26.9; 36.4	32.2	27.4; 36.9	36.2	31.3; 41.1
Yes	26.8	19.5; 34.0	28.9	21.4; 36.3	44.4	36.2; 52.5
Sexual partnership						
Casual	35.4	29.9; 41.0	28.8	23.5; 34.0	35.8	30.2; 41.4
Steady	23.8	18.2; 29.3	34.4	28.2; 40.5	41.9	35.4; 48.3
Comorbidity						
No	29.1	25.1; 33.2	31.9	27.7; 36.0	39.0	34.6; 43.4
Yes	45.7	29.2; 62.2	22.9	8.9; 36.8	31.4	16.0; 46.8
Mental disorder						
No	30.7	26.4; 35.1	31.2	26.8; 35.5	38.1	33.5; 42.7
Yes	27.8	18.0; 37.7	31.6	21.4; 41.9	40.5	29.7; 51.3
Psychotropic medication use						
No	30.4	26.1; 34.7	31.1	26.8; 35.4	38.5	34.0; 43.0
Yes	29.4	18.6; 40.2	32.4	21.2; 43.5	38.2	26.7; 49.8
Service entry						
New case	32.8	28.2; 37.3	31.5	27.0; 36.0	35.7	31.1; 40.4
Transferred case	21.1	13.4; 28.8	30.3	21.6; 38.9	48.6	39.2; 58.0
First VL result						
Suppressed	17.1	5.6; 28.6	34.1	19.6; 48.7	48.8	33.5; 64.1
Not suppressed	33.6	29.1; 38.2	32.2	27.8; 36.7	34.1	29.6; 38.6
Undetectable	16.2	4.3; 28.1	27.0	12.7; 41.3	56.8	40.8; 72.7
No information	0.0	0.0; 0.0	0.0	0.0; 0.0	100.0	100.0; 100.0
First CD4+ result						
<200 cells/mm^3^	33.3	20.4; 46.3	27.5	15.2; 39.7	39.2	25.8; 52.6
200-350 cells/mm^3^	37.1	27.0; 47.1	33.7	23.9; 43.5	29.2	19.8; 38.7
>350 cells/mm^3^	29.2	24.5; 33.9	32.2	27.4; 37.0	38.6	33.6; 43.6
No information	0.0	0.0; 0.0	0.0	0.0; 0.0	100.0	100.0; 100.0
HIV staging						
Stage I	31.5	25.7; 37.4	30.3	24.5; 36.1	38.2	32.0; 44.3
Stage II	24.6	16.9; 32.2	36.1	27.5; 44.6	39.3	30.7; 48.0
Stage III	36.8	26.6; 46.9	33.3	23.4; 43.2	29.9	20.3; 39.5
Stage IV	33.3	20.4; 46.3	27.5	15.2; 39.7	39.2	25.8; 52.6
No information	0.0	0.0; 0.0	0.0	0.0; 0.0	100.0	100.0; 100.0
ART scheme						
3TC+TDF+DTG	33.9	29.4; 38.3	33.6	29.2; 38.1	32.5	28.1; 36.9
3TC+TDF+EFZ	10.0	0.7; 19.3	27.5	13.7; 41.3	62.5	47.5; 77.5
Other	14.3	-4.0; 32.6	7.1	-6.3; 20.6	78.6	57.1; 100.1
Did not start ART	0.0	0.0; 0.0	0.0	0.0; 0.0	100.0	100.0; 100.0
Side effect						
No	32.0	27.5; 36.4	33.4	28.9; 37.9	34.6	30.1; 39.2
Yes	30.0	19.3; 40.7	28.6	18.0; 39.2	41.4	29.9; 53.0
Did not start ART	0.0	0.0; 0.0	0.0	0.0; 0.0	100.0	100.0; 100.0
Number of regimens						
1	32.8	28.5; 37.2	33.3	28.9; 37.6	33.9	29.5; 38.3
>1	17.4	6.4; 28.3	23.9	11.6; 36.2	58.7	44.5; 72.9
Did not start ART	0.0	0.0; 0.0	0.0	0.0; 0.0	100.0	100.0; 100.0
Last CD4+ count result						
<200 cells/mm^3^	34.8	15.3; 54.2	13.0	-0.7; 26.8	52.2	31.8; 72.6
200-350 cells/mm^3^	31.9	18.6; 45.2	25.5	13.1; 38	42.6	28.4; 56.7
>350 cells/mm^3^	30.7	26.3; 35.1	33.7	29.3; 38.2	35.6	31.1; 40.1
No information	0.0	0.0; 0.0	0.0	0.0; 0.0	100.0	100.0; 100.0
Last VL result						
Suppressed	24.4	11.2; 37.5	22.0	9.3; 34.6	53.7	38.4; 68.9
Not suppressed	17.9	9.4; 26.5	14.1	6.4; 21.8	67.9	57.6; 78.3
Undetectable	34.4	29.6; 39.2	36.7	31.9; 41.6	28.9	24.3; 33.4
No information	0.0	0.0; 0.0	0.0	0.0; 0.0	100.0	100.0; 100.0
Opportunistic infection						
No	25.5	16.7; 34.3	29.8	20.5; 39.0	44.7	34.6; 54.7
Yes	31.9	27.4; 36.4	32.1	27.6; 36.6	36.0	31.4; 40.7
No information	0.0	0.0; 0.0	0.0	0.0; 0.0	100.0	100.0; 100.0
Other STI						
No	28.7	23.2; 34.1	29.1	23.6; 34.5	42.3	36.3; 48.2
Yes	32.8	26.9; 38.7	34.4	28.4; 40.4	32.8	26.9; 38.7
No information	0.0	0.0; 0.0	0.0	0.0; 0.0	100.0	100.0; 100.0
Active search performed						
No	18.2	-4.6; 41.0	54.5	25.1; 84.0	27.3	1.0; 53.6
Yes	30.5	26.5; 34.6	30.7	26.7; 34.8	38.7	34.5; 43.0
Time between diagnosis and ART						
<50 days	37.8	32.0; 43.6	28.5	23.1; 33.9	33.7	28.1; 39.3
50-100 days	28.3	20.5; 36.2	40.2	31.6; 48.7	31.5	23.4; 39.6
>100 days	16.7	9.2; 24.1	33.3	23.9; 42.8	50.0	40.0; 60.0
Did not start ART	5.3	-4.8; 15.3	0.0	0.0; 0.0	94.7	84.7; 104.8
No appointments						
No	52.9	43.3; 62.5	27.9	19.3; 36.5	19.2	11.7; 26.8
Yes	24.5	20.3; 28.7	32.1	27.6; 36.6	43.4	38.6; 48.2

*IR - incidence rate; 95%CI - 95% Confidence Interval; VL - viral load; ART - antiretroviral therapy; STI - sexually transmitted infection; 3TC+TDF+EFZ - lamivudine+tenofovir+efavirenz; 3TC+TDF+DTG - lamivudine+tenofovir+dolutegravir.*

In bivariate analysis with Poisson Regression with robust variance, 25 variables showed statistical significance (p<0.25), which were used to construct multiple models, both for the association of poor adherence and loss of follow-up, as shown in Table 2.

**Table 2:** Univariate analysis of factors associated with poor adherence and loss to follow-up among adolescents and young people living with HIV, Maringá, Paraná, Brazil, 2025

Variable	Poor adherence			Loss to follow-up		
	RR	95%CI	*p* value	RR	95%CI	*p* value
Sex						
Female	Reference		0.099	Reference		<0.001
Male	0.76	0.55; 1.05		0.64	0.52; 0.79	
Age group						
15-19 years	Reference		0.018	Reference		0.326
20-24 years	0.74	0.58; 0.95		0.87	0.66; 1.14	
Marital status						
No partner	Reference		0.779	Reference		0.013
With partner	1.05	0.73; 1.49		1.34	1.06; 1.69	
Race/color						
White	Reference		0.107	Reference		0.045
Non-white	1.20	0.96; 1.52		1.23	1.00; 1.52	
Education						
≤12 years	Reference		0.065	Reference		0.005
>12 years	0.80	0.64; 1.01		0.72	0.57; 0.90	
Religion						
No	Reference		0.601	Reference		0.051
Yes	1.06	0.85; 1.31		1.22	0.99; 1.49	
Sexual orientation						
Heterosexual	Reference		0.279	Reference		<0.001
Homosexual	0.89	0.63; 1.26		0.65	0.53; 0.79	
Bisexual	1.12	0.74; 1.70		0.58	0.35; 0.94	
Occupation						
Employed/self-employed	Reference		0.385	Reference		0.008
Student/houseperson	1.20	0.91; 1.59		1.05	0.76; 1.45	
Unemployed	0.80	0.27; 2.36		1.55	1.17; 2.04	
Distance to service						
<5 km	Reference		0.154	Reference		0.007
5-10 km	0.81	0.63; 1.03		1.06	0.80; 1.41	
>10 km	1.01	0.76; 1.35		1.44	1.08; 1.92	
Homeless person						
No	Reference		-	Reference		0.901
Yes	-	-		0.93	0.35; 2.51	
Person deprived of liberty						
No	Reference		0.979	Reference		0.235
Yes	0.98	0.24; 3.90		1.41	0.79; 2.51	
Alcohol use						
No	Reference		0.588	Reference		0.165
Yes	0.94	0.75; 1.17		0.86	0.70; 1.06	
Tobacco use						
No	Reference		0.647	Reference		0.099
Yes	0.94	0.73; 1.20		1.18	0.96; 1.45	
Drug use						
No	Reference		0.845	Reference		0.197
Yes	1.02	0.80; 1.31		1.15	0.93; 1.42	
Sexual partner						
No	Reference		0.013	Reference		0.016
Yes	1.31	1.05; 1.62		1.27	1.04; 1.56	
Comorbidity						
No	Reference		0.162	Reference		0.295
Yes	0.66	0.37; 1.17		0.78	0.49; 1.23	
Mental disorder						
No	Reference		0.608	Reference		0.284
Yes	1.07	0.80; 1.44		1.14	0.89; 1.47	
Psychotropic use						
No	Reference		0.704	Reference		0.592
Yes	1.06	0.78; 1.44		1.08	0.81; 1.42	
Service entry						
New case	Reference		0.161	Reference		0.002
Transferred case	1.19	0.93; 1.53		1.37	1.21; 1.68	
First VL result						
Suppressed	Reference		0.112	Reference		<0.001
Unsuppressed	0.65	0.53; 1.02		0.68	0.52; 0.89	
Undetectable	0.93	0.57; 1.52		1.06	0.77; 1.47	
First CD4+ result						
<200 cells/mm^3^	Reference		0.633	Reference		0.280
200-350 cells/mm^3^	1.05	0.66; 1.61		0.81	0.53; 1.25	
>350 cells/mm^3^	1.16	0.77; 1.75		1.05	0.76; 1.46	
HIV staging						
Stage I	Reference		0.348	Reference		0.319
Stage II	1.20	0.94; 1.54		1.13	0.90; 1.43	
Stage III	0.96	0.70; 1.31		0.82	0.59; 1.14	
Stage IV	0.91	0.60; 1.39		0.99	0.70; 1.39	
ART regimen						
3TC+TDF+DTG	Reference		0.060	Reference		<0.001
3TC+TDF+EFZ	1.46	1.05; 2.03		1.78	1.47; 2.14	
Other	0.66	0.13; 3.31		1.74	1.34; 2.26	
Side effect						
No	Reference		0.898	Reference		0.418
Yes	0.97	0.70; 1.36		1.11	0.85; 1.45	
Number of regimens						
1	Reference		0.324	Reference		<0.001
>1	1.21	0.82; 1.78		1.58	1.28; 1.94	
Latest CD4+ result						
<200 cells/mm^3^	Reference		0.324	Reference		0.844
200-350 cells/mm^3^	1.63	0.56; 4.67		0.96	0.59; 1.56	
>350 cells/mm^3^	1.92	0.72; 5.08		0.90	0.60; 1.35	
Latest VL result						
Deleted	Reference		0.741	Reference		<0.001
Not suppressed	0.92	0.48; 1.77		1.21	0.89; 1.64	
Undetectable	1.09	0.67; 1.78		0.70	0.51; 0.96	
Opportunistic infection						
No	Reference		0.640	Reference		0.098
Yes	0.93	0.70; 1.23		0.82	0.65; 1.03	
Other STI						
No	Reference		0.919	Reference		0.055
Yes	1.01	0.81; 1.25		0.81	0.66; 1.00	
Active search performed						
No	Reference		0.060	Reference		0.901
Yes	0.67	0.44; 1.01		1.06	0.39; 2.85	
Time between diagnosis and ART						
<50 days	Reference		0.002	Reference		<0.001
50-100 days	1.63	1.06; 1.74		1.13	0.86; 1.46	
>100 days	1.55	1.19; 2.01		1.59	1.28; 1.96	
No appointments						
No	Reference		0.003	Reference		<0.001
Yes	1.62	1.18; 2.22		2.34	1.57; 3.48	

*RR - relative risk; 95%CI - 95% Confidence Interval; VL - viral load; ART - antiretroviral therapy; STI - sexually transmitted infection; 3TC+TDF+EFZ - lamivudine+tenofovir+efavirenz; 3TC+TDF+DTG - lamivudine+tenofovir+dolutegravir.*

*Note: the hyphen symbol indicates that, for that subvariable, it was not possible to obtain the parameter for the model due to the absence of observations.*

In the final multivariate model of poor adherence presented in Table 3, there was evidence of negatively associated factors, which included having a sexual partner, time between diagnosis and ART initiation ranging from 50-100 days and >100 days, and absence from appointments. On the other hand, the only positively associated factor was the age group of 20-24 years.

**Table 3:** Multivariate analysis of factors associated with poor treatment adherence among adolescents and young people living with HIV, Maringá, Paraná, Brazil, 2025

Variable		Poor adherence^*^	
	RR	95%CI	*p* value
Age group			
15-19 years		Reference	0.040
20-24 years	0.77	0.61; 0.98	
Sexual partner			
No		Reference	0.027
Yes	1.26	1.02; 1.56	
Time between diagnosis and ART			
<50 days		Reference	0.016
50-100 days	1.27	1.01; 1.62	
>100 days	1.45	1.11; 1.89	
Absence from appointment			
No		Reference	0.007
Yes	1.52	1.12; 2.06	

RR - relative risk; ART - antiretroviral therapy; 95%CI - 95% Confidence Interval;

* final multiple model adjusted for all remaining variables and for the “distance to service” variable.

In the final multivariate model for loss to follow-up presented in Table 4, the negatively associated factors were drug use, an ART regimen consisting of lamivudine+tenofovir+efavirenz and another regimen, the last unsuppressed viral load result before the outcome, time between diagnosis and ART of 50-100 days and >100 days, as well as absence from an appointment before loss to follow-up. Positively associated factors were alcohol use and the first unsuppressed viral load test, and the last undetectable viral load test.

**Table 4:** Multivariate analysis of factors associated with loss to follow-up among adolescents and young adults living with HIV, Maringá, Paraná, Brazil, 2025

Variable		Loss to follow-up^*^	
	RR	95%CI	*p* value
Alcohol use			
No		Reference	0.021
Yes	0.80	0.66; 0.96	
Drug use			
No		Reference	0.026
Yes	1.25	1.02; 1.52	
First VL test result			
Suppressed		Reference	0.002
Not suppressed	0.73	0.54; 1.00	
Undetectable	1.20	0.86; 1.66	
ART regimen			
3TC+TDF+DTG		Reference	<0.001
3TC+TDF+EFZ	1.46	1.18; 1.80	
Other	1.63	1.27; 2.09	
Last VL test result			
Suppressed		Reference	<0.001
Not suppressed	1.12	0.83; 1.53	
Undetectable	0.66	0.49; 0.91	
Time between diagnosis and ART			
<50 days		Reference	0.002
50-100 days	1.15	0.90; 1.47	
>100 days	1.46	1.18; 1.79	
Absence from appointment			
No		Reference	0.002
Yes	1.84	1.24; 2.71	

RR - relative risk; 95%CI - 95% Confidence Interval; VL - viral load; ART - antiretroviral therapy; 3TC+TDF+EFZ - lamivudine+tenofovir+efavirenz; 3TC+TDF+DTG - lamivudine+tenofovir+dolutegravir;

* final multiple model adjusted for all remaining variables and for the “distance to service” variable.

## DISCUSSION

The study found that treatment failure rates among adolescents and young PLHIV, determined by poor adherence and loss to follow-up, were 78.4% and 68.3%, respectively. To address these challenges, efforts are needed to mitigate and understand risk factors and thus improve treatment adherence.

As noted, more than two-thirds of adolescents and young adults undergoing treatment did not fully adhere to ART at some point during follow-up. This finding exceeds the 5% threshold established by the World Health Organization (WHO)^(14)^. Although medications are free, financially disadvantaged users face difficulties in accessing care due to transportation costs, and loss of income due to time off work and other logistical challenges^(15)^.

Adolescents aged 15 to 19 are increasingly sexually active. This raises concerns about inadvertently revealing their status to a sexual partner if they are seen at HIV testing sites or taking antiretroviral medication^(15)^. This context makes sexual partnership a factor for poor adherence, due to fear of revealing the diagnosis, stigma, and prejudice, consequently leading to negative repercussions on treatment adherence.

The use of illicit drugs has been consistently identified as a factor for loss to follow-up, although the specific type of substance used by these adolescents and young people is not always reported. A study with Brazilian users of Hornet (an app and social network for gay men) indicates that, among sexual minorities, the association between cannabis use and ART adherence has not yet been evidenced, while the use of cocaine, heroin, and methamphetamines is negatively associated with adherence^(16)^. Thus, screening for substance use during HIV treatment has proven effective in identifying disorders and referring patients to appropriate treatment^(16)^.

As for the ART regimen, it was observed that combinations based on efavirenz and other antiretrovirals, except dolutegravir, represented a greater propensity for viral load failure. The study highlights the benefits of the efavirenz regimen in adherence and virological suppression^(17)^. It is worth noting that the cohort began in 2017, when the WHO approved the transition from the efavirenz regimen to dolutegravir, indicating the need for further studies exploring this association.

When there is poor treatment adherence, the viral load tends to remain elevated. From this perspective, the last unsuppressed viral load result is negatively associated with the outcome of loss to follow-up. This predictor may be related to patients’ low income, which compromises their healthcare and requires travel to obtain medication, resulting in missed doses and a higher risk of virological failure^(18)^. A study conducted in Zimbabwe found that low income was associated with high viral load (≥1,000 copies/mL) in patients on ART^(19)^.

A time between diagnosis and ART of 50-100 days and >100 days was a factor for both poor adherence and loss to follow-up. This result is noteworthy, since the WHO recommends starting ART on the same day of diagnosis^(20)^. However, it was shown that loss-to-follow-up rates have been higher in developing countries^(21)^. Additionally, the International Society for Antiretrovirals also specifically recommends starting ART within 14 days for PLHIV who have opportunistic infections^(22)^. A study conducted in Pattaya and Bangkok identified that initiating ART within five days of HIV infection increased the likelihood of having undetectable HIV DNA on CD4+ cells^(23)^.

Therefore, early diagnosis of HIV and prompt initiation of treatment are essential as a beneficial measure for individuals’ health and for society, as they contribute to preventing transmission. Routine testing for sexually transmitted infections plays an essential role in identifying and treating many infections that would otherwise go undetected and untreated in asymptomatic individuals^(24)^.

Patients with a history of missed appointments tend to have poor adherence and are lost to follow-up. For instance, a study in Ethiopia identified that adolescents and young adults who missed their appointments were more likely to miss their doses and increased their chances of virological failure^(25)^.

In the present study, being between 20 and 24 years old was a protective factor for poor adherence, which differs from what was observed in other studies^(15,26)^. As we age, the perception of HIV-related stigma can reduce our willingness to disclose our status and utilize healthcare services. Furthermore, psychological, physical, cognitive, and even sexual fatigue can affect treatment adherence^(15)^.

The current study results also diverge from the literature regarding the association between alcohol use and loss to follow-up. The literature shows that those with a history of alcohol use are more likely to miss clinical appointments and not adhere to ART. Furthermore, alcohol use is not only associated with treatment failure but is also considered a major risk factor for morbidity and mortality in Latin America^(16,27)^. The relationship between alcohol use and loss to follow-up in the Brazilian context deserves further exploration, since, in this study, alcohol use was positively associated with loss to follow-up.

The first non-suppressed viral load test was a protective factor for loss of follow-up, making the adolescent and/or young person see the need to continue treatment to achieve an undetectable viral load^(28)^. Even when the result of the last viral load test becomes undetectable, young people who are lost to follow-up see the need to maintain HIV care in order to have a good quality of life.

Therefore, healthcare professionals should improve their interactions with PLHIV in order to raise awareness of the U=U concept, meaning undetectable equals untransmissible, a scientific expression meaning that PLHIV undergoing treatment will not transmit the virus. A better understanding of this concept has the potential to challenge stigma and, in turn, support greater HIV treatment adherence^(29)^.

### Study limitations

Since this is an ambispective cohort study, in which patient information was extracted from their routine medical records and medication dispensing records, data completeness depends on the robustness of the data collection performed by the outpatient clinic staff and is therefore subject to errors in data recording. Nevertheless, the results may inform the development of strategies aimed at improving care for adolescents and young PLHIV.

Furthermore, the sample included all adolescents and young PLHIV treated at the outpatient clinic, which provides services to 30 municipalities, reducing potential selection bias and ensuring internal validity, considering the quality of the evidence. Concerning external validity, the study allows for generalizability of results to contexts similar to the service where this research was conducted. However, multicenter studies in Brazil are recommended to obtain a national overview.

### Contributions to nursing, health, or public policy

The results of this study have the potential to contribute to public health by enabling the identification of situations of vulnerability and the determinants associated with poor adherence and loss to follow-up in HIV treatment among adolescents and young people. Thus, they can support the reformulation of health policies aimed at improving services and care strategies, contributing to achieving the goal of eliminating AIDS by 2030.

## CONCLUSIONS

The incidence of poor adherence and loss to follow-up among adolescents and young PLHIV is high and is mainly associated with socioeconomic and clinical-epidemiological factors. These results reiterate the various determinants intertwined with the initiation and treatment of this condition, and point to the need for strategies aimed at adherence to and retention in HIV care among the groups most vulnerable to these outcomes, such as adolescents and young people.

## Data Availability

The research data are available only upon request.
