# Supplementary material for: Treatment failure in adolescents and young people living with HIV: a cohort study
Source: Rev Bras Enferm. 2026 Jun 22;79:e20250340. doi: 10.1590/0034-7167-2025-0340 (PMC13321819; doi:10.1590/0034-7167-2025-0340)
Supplement: Translation

# Translation: Insucesso do tratamento de adolescentes e jovens vivendo com HIV: estudo de coorte

**DOI:** 10.1590/0034-7167-2025-0340pt

**Authors:** Camila Moraes Garollo Piran, Alana Vitória Escritori Cargnin, Mariana Martire Mori, Marcelo da Silva, Sonia Silva Marcon, Ieda Harumi Higarashi, João Manuel Graça Frade, Marcela Demitto Furtado

**Affiliations:** I Universidade Estadual de Maringá. Maringá, Paraná, Brasil; II Instituto Politécnico de Leiria. Leiria, Portugal

**Keywords:** HIV, Síndrome da Imunodeficiência Humana, Perda de Seguimento, Adolescente, Adulto Jovem.

## Abstract

**Objetivos:**

analisar os fatores associados à má adesão e perda de seguimento entre adolescentes e jovens vivendo com HIV.

**Métodos:**

coorte ambispectiva de 512 adolescentes e jovens vivendo com HIV em tratamento no serviço especializado no noroeste do Paraná, Brasil. Realizou-se Regressão de Poisson com variância robusta.

**Resultados:**

ter parceria sexual, tempo entre diagnóstico e início da terapia antirretroviral, e ausência em consultas se associou negativamente à má adesão. Faixa etária de 20-24 anos se associou positivamente. Uso de drogas, esquema de terapia antirretroviral composto por lamivudina+tenofovir+efavirenz, último resultado de carga viral não suprimido antes do desfecho, tempo entre diagnóstico e terapia antirretroviral, e ausência em consulta se associaram à perda de seguimento. Uso de álcool, primeiro exame de carga viral não suprimido e último exame de carga viral indetectável foram positivamente associados.

**Conclusões:**

reiteraram-se os diversos determinantes imbricados ao adoecimento e tratamento entre adolescentes e jovens vivendo com HIV.

## INTRODUÇÃO

O vírus da imunodeficiência humana (HIV) continua sendo uma infecção desafiadora que repercute de forma significativa nos sistemas de saúde, especialmente devido à progressão do HIV para a Síndrome da Imunodeficiência Humana Adquirida (aids) e sua morbimortalidade^(1)^.

Apesar dos benefícios da terapia antirretroviral (TARV), o relatório global da *Join United Nations Program on HIV/AIDS* de 2023 revelou que, das 39,9 milhões de pessoas que vivem com HIV (PVHIV) em todo o mundo, apenas 77% estão em uso da TARV^(2)^. No mundo, a incidência do HIV entre adolescentes e jovens tem sido de 22,7 por 100.000 habitantes (Intervalo de Confiança de 95% (IC95%): 3,1 a -2,0)^(3)^. Estima-se que, de sete novas infecções por HIV, duas delas ocorrem entre adolescentes e jovens de 15 a 24 anos^(4)^.

Adolescentes e jovens vivendo com HIV frequentemente apresentam resultados adversos no tratamento, como má adesão e perda de acompanhamento em diferentes localidades^(5-7)^. Considerando a magnitude da problemática, há um esforço global para acabar com a epidemia de HIV até 2030, constituindo, inclusive, uma das metas dos Objetivos de Desenvolvimento Sustentável da Organização das Nações Unidas^(8)^.

Contudo, no caso da infecção pelo HIV, assim como no caso de doenças crônicas, o indivíduo desempenha papel fundamental no cuidado com a própria saúde, e por isso deve assumir a responsabilidade pelo seu autocuidado. No contexto do HIV, a adesão ao tratamento é indispensável para resultados bem-sucedidos, e esta, por sua vez, é influenciada pela história do paciente, suas motivações e seu comprometimento com a própria qualidade de vida^(9)^.

No entanto, gerenciar o tratamento entre adolescentes e jovens é desafiador, especialmente devido a fatores como imaturidade, regime terapêutico, acesso ao serviço de saúde, baixa condição socioeconômica acentuada pela doença e estigma social^(10,11)^.

Em revisão de escopo conduzida pelos autores, foi identificado que vários fatores intrínsecos e extrínsecos interferem no acompanhamento de saúde de adolescentes e jovens vivendo com HIV; porém, ainda é insuficiente. Apesar da variedade de estudos, surgiu a necessidade de abordar algumas lacunas, utilizando coorte ambispectiva como método de estudo e articulação de dados voltados aos seus determinantes sociais de saúde, os quais podem estar relacionados ao insucesso do tratamento.

## OBJETIVOS

Analisar os fatores associados à má adesão e perda de seguimento entre adolescentes e jovens vivendo com HIV.

## MÉTODOS

### Aspectos éticos

O estudo foi desenvolvido em consonância com as diretrizes estabelecidas pelas Resolução nº 466/12 e n^o^ 510/16 do Conselho Nacional de Saúde, sendo aprovado pelo Comitê de Ética em Pesquisa com Seres Humanos. Os registros contendo a identificação dos participantes foram substituídos por códigos e números, para garantir o anonimato e preservar a identidade dos mesmos.

### Desenho, período e local do estudo

Estudo epidemiológico, do tipo coorte aberta ambispectiva com adolescentes e jovens vivendo com HIV. Para guiar o estudo de acordo com as recomendações da EQUATOR *network*, utilizou-se o *STrengthening the Reporting of OBservational studies in Epidemiology*.

O estudo foi conduzido no ambulatório de IST/HIV/aids e hepatites virais localizado na região Noroeste do estado do Paraná, Brasil. Nesse ambulatório, são oferecidos três serviços distintos: Centro de Testagem e Aconselhamento; Serviço de Atenção Especializada; e Unidade Dispensadora de Medicamentos.

### População ou amostra; critérios de inclusão e exclusão

A amostra foi não probabilística (consecutiva), composta por todos os adolescentes e jovens com Classificação Internacional de Doenças - indicador de estatísticas dos agravos de mortalidade e morbidade - B20.0 a B24 referente ao HIV/aids.

Adotou-se como critério de elegibilidade ser adolescente e jovem na faixa etária entre 10 e 24 anos atendidos que procuraram o ambulatório de referência para realização do acompanhamento clínico e medicamentoso entre 01 de janeiro de 2017 e 31 de dezembro de 2023. Só foram incluídos os casos de transmissão por via sexual. O ano de 2017 foi definido devido ao fato de que, em 07 de junho de 2016, na Declaração Política da Assembleia Geral das Nações Unidas sobre o Fim da Aids, os países se comprometeram a acelerar a resposta para lutar contra o HIV e acabar com a epidemia de aids até 2030^(12)^. A inclusão de participantes até 31 de dezembro de 2023, por sua vez, justifica-se pela necessidade de acompanhar o caso de HIV/aids por um período de tempo até o desfecho ou até 24 anos, 11 meses e 29 dias, tempo suficiente para investigação da adesão, má adesão ou perda de seguimento. Foram excluídos do estudo 21 participantes, sendo 19 jovens que tiveram diagnóstico aos 24 anos, mas se cadastraram no serviço com 25 anos, além de dois casos por óbitos.

### Protocolo do estudo

Os dados foram coletados entre junho de 2024 e janeiro de 2025 mediante consulta ao prontuário dos pacientes atendidos no ambulatório de IST/HIV/aids e hepatites virais. A pesquisadora responsável, juntamente com a equipe de pesquisa (uma doutoranda e uma mestranda), realizou o agendamento prévio dos dias e horários junto ao serviço, para não interferir na rotina do setor. Foi utilizado instrumento estruturado para obtenção das variáveis do estudo. O referido instrumento foi elaborado conforme as informações dos protocolos do Ministério da Saúde, o qual foi submetido a um processo de avaliação simples por oito especialistas para posterior início da coleta de dados.

As variáveis dependentes consideradas para o estudo foram má adesão e perda de seguimento. No caso de tratamento de HIV, consideram-se adesão o comparecimento às consultas agendadas, a realização de exames de controle solicitados e a retirada dos medicamentos na data prevista. Por conseguinte, má adesão é a não retirada de TARV após sete dias da data prevista. Abandono/perda de seguimento se referem aos casos de não retirada de medicações antirretrovirais junto à farmácia do serviço, a partir de três meses após a data prevista e/ou não retorno às consultas em seis meses^(13)^. As variáveis independentes foram agrupadas em eixos temáticos: sociodemográficas; comportamentais; clínicas, imunológicas e laboratoriais.

### Análise dos resultados e estatística

Foram estimadas as taxas de incidência para adesão, má adesão e perda de seguimento, estratificadas por variáveis individuais e de tratamento. O cálculo se deu utilizando a proporção das observações de cada subvariável naquele desfecho em relação ao total de observações para aquela característica, e seu resultado foi expresso em 100. Essas taxas foram acompanhadas por IC95%. A análise descritiva também incluiu o tempo médio (TM) de seguimento para cada desfecho, com os respectivos desvios padrão (DPs).

Em seguida, foram construídos modelos de Regressão de Poisson com variância robusta para verificar a associação das variáveis do estudo com os desfechos “má adesão” e “perda de seguimento”, usando-se a “adesão” como categoria de referência na comparação. A análise bivariada considerou as variáveis com valor de p<0,25 no teste qui-quadrado de Wald para posterior inclusão nos modelos múltiplos. As variáveis marginalmente significativas na análise bivariada foram avaliadas quanto à multicolinearidade antes da etapa seguinte.

A construção dos modelos múltiplos para cada um dos desfechos considerou o método de seleção *stepwise backward*, no qual as variáveis selecionadas na análise bivariada foram inseridas conjuntamente e, então, removidas uma a uma, conforme a significância estatística. O modelo final de cada desfecho (“má adesão” e “perda de seguimento”) foi ajustado para as variáveis remanescentes (p<0,05), bem como para a variável “distância até serviço”, por entender-se que o deslocamento até o ambulatório poderia influenciar o seguimento.

Os riscos relativos (RR), acompanhados por seus IC95%, foram utilizados como medidas de associação entre as variáveis independentes e dependentes, permitindo avaliar sua magnitude e sua direção. Importante pontuar que as variáveis com informações faltantes foram incluídas na análise descritiva, mas não integraram os modelos de regressão, visto que poderiam comprometer a integridade e fidedignidade da análise, impedindo uma interpretação apropriada. Todas as análises foram realizadas no *software Statistical Package for the Social Sciences* ^®^, versão 21.0.

## RESULTADOS

No período em estudo, 512 adolescentes e jovens vivendo com HIV atenderam aos critérios de elegibilidade do estudo, sendo 155 classificados como aderentes, 160, má aderentes, e 197, em perda de seguimento. O tempo mediano do acompanhamento do paciente adolescente em adesão foi de 807,6 dias (DP=617,4), enquanto que, de má adesão, foi de 779,6 dias (DP=785,5), e a de perda de seguimento foi de 755,1 dias (DP=595,8). Em relação ao jovem, o TM de adesão foi de 538,4 dias (DP=431,5), o de má adesão, de 454,7 dias (DP=396,0), e o de perda de seguimento, de 501,8 (DP=458,4).

Em relação às principais características dos participantes, no grupo de adesão, a taxa de incidência maior foi entre o sexo masculino (32%), jovens com idade entre 20 e 24 anos (31,7%), raça/cor branca (32,8%) e com mais de 12 anos de estudos (38,3%). Referente ao grupo de má adesão, observou-se predomínio do sexo masculino (31,8%), grupo etário entre 15 e 19 anos (40,6%) e raça/cor não branca (32,8%). Já no grupo de perda de seguimento, predominaram o sexo feminino (57,1%), faixa etária de 20 a 24 anos (38,6%), raça/cor não branca (44,015) e escolaridade <12 anos (43%). Outras características dos adolescentes e jovens vivendo com HIV podem ser observadas na Tabela 1.

**Tabela 1:** Taxas de incidência de adesão, má adesão e perda de seguimento entre adolescentes e jovens vivendo com HIV, Maringá, Paraná, Brasil, 2025

Variável	Adesão		Má adesão		Perda de seguimento	
	TI	IC95%	TI	IC95%	TI	IC95%
Sexo						
Feminino	16,1	6,5; 25,7	26,8	15,2; 38,4	57,1	44,2; 70,1
Masculino	32,0	27,7; 36,3	31,8	27,5; 36,1	36,2	31,8; 40,6
Faixa etária						
15-19 anos	21,6	12,2; 31,0	40,6	29,4; 51,7	37,8	26,8; 48,9
20-24 anos	31,7	27,4; 36,1	29,7	25,4; 34,0	38,6	34,0; 43,1
Estado civil						
Sem companheiro	31,3	27,0; 35,6	32,0	27,7; 36,3	36,7	32,2; 41,1
Com companheiro	22,6	12,2; 33,0	25,8	14,9; 36,7	51,6	39,2; 64,1
Raça/cor						
Branca	32,8	28,1; 37,5	30,7	26,0; 35,3	36,5	31,7; 41,4
Não branca	23,1	16,0; 30,3	32,8	24,9; 40,8	44,0	35,6; 52,4
Escolaridade						
≤12 anos	25,3	20,5; 30,1	31,6	26,5; 36,8	43,0	37,6; 48,5
>12 anos	38,3	31,5; 45,1	30,6	24,2; 37,1	31,1	24,6; 37,6
Religião						
Não	33,2	27,7; 38,7	32,5	27,1; 38,0	34,3	28,7; 39,8
Sim	26,6	20,9; 32,4	29,7	23,8; 35,6	43,7	37,2; 50,1
Orientação sexual						
Heterossexual	18,8	10,2; 27,3	21,3	12,3; 30,2	60,0	49,3; 70,7
Homossexual	32,9	28,2; 37,6	31,6	27,0; 36,2	35,5	30,7; 40,3
Bissexual	28,3	15,2; 41,3	45,7	31,3; 60,0	26,1	13,4; 38,8
Ocupação						
Empregado/autônomo	31,3	27,0; 35,7	30,9	26,5; 35,2	37,8	33,2; 42,3
Estudante/do lar	26,7	15,5; 37,9	40,0	27,6; 52,4	33,3	21,4; 45,3
Desempregado	16,7	-0,6; 33,9	11,1	-3,4; 25,6	72,2	51,5; 92,9
Distância até serviço						
<5 km	29,9	22,3; 37,6	38,7	30,5; 46,8	31,4	23,6; 39,2
5-10 km	34,4	28,6; 40,1	28,6	23,2; 34,1	37,0	31,2; 42,9
>10 km	21,4	13,8; 29,0	28,6	20,2; 36,9	50,0	40,7; 59,3
Sem informação	0,0	0,0; 0,0	0,0	0,0; 0,0	100,0	100,0; 100,0
Pessoa em situação de rua						
Não	30,2	26,2; 34,2	31,6	27,5; 35,6	38,3	34,0; 42,5
Sim	40,0	-2,9; 82,9	0,0	0,0; 0,0	60,0	17,1; 102,9
Pessoa privada de liberdade						
Não	30,4	26,4; 34,4	31,4	27,3; 35,4	38,3	34,0; 42,5
Sim	20,0	-15,1; 55,1	20,0	-15,1; 55,1	60,0	17,1; 102,9
Uso de álcool						
Não	27,8	21,7; 33,8	31,1	24,8; 37,4	41,1	34,5; 47,8
Sim	32,0	26,8; 37,3	31,4	26,1; 36,6	36,6	31,2; 42,1
Uso de tabaco						
Não	31,7	26,8; 36,6	33,7	28,7; 38,7	34,6	29,6; 39,6
Sim	27,4	20,6; 34,1	26,2	19,5; 32,8	46,4	38,9; 54,0
Uso de drogas						
Não	31,6	26,9; 36,4	32,2	27,4; 36,9	36,2	31,3; 41,1
Sim	26,8	19,5; 34,0	28,9	21,4; 36,3	44,4	36,2; 52,5
Parceria sexual						
Casual	35,4	29,9; 41,0	28,8	23,5; 34,0	35,8	30,2; 41,4
Fixa	23,8	18,2; 29,3	34,4	28,2; 40,5	41,9	35,4; 48,3
Comorbidade						
Não	29,1	25,1; 33,2	31,9	27,7; 36,0	39,0	34,6; 43,4
Sim	45,7	29,2; 62,2	22,9	8,9; 36,8	31,4	16,0; 46,8
Transtorno mental						
Não	30,7	26,4; 35,1	31,2	26,8; 35,5	38,1	33,5; 42,7
Sim	27,8	18,0; 37,7	31,6	21,4; 41,9	40,5	29,7; 51,3
Uso de psicotrópico						
Não	30,4	26,1; 34,7	31,1	26,8; 35,4	38,5	34,0; 43,0
Sim	29,4	18,6; 40,2	32,4	21,2; 43,5	38,2	26,7; 49,8
Entrada no serviço						
Caso novo	32,8	28,2; 37,3	31,5	27,0; 36,0	35,7	31,1; 40,4
Caso transferido	21,1	13,4; 28,8	30,3	21,6; 38,9	48,6	39,2; 58,0
Primeiro resultado de CV						
Suprimido	17,1	5,6; 28,6	34,1	19,6; 48,7	48,8	33,5; 64,1
Não suprimido	33,6	29,1; 38,2	32,2	27,8; 36,7	34,1	29,6; 38,6
Indetectável	16,2	4,3; 28,1	27,0	12,7; 41,3	56,8	40,8; 72,7
Sem informação	0,0	0,0; 0,0	0,0	0,0; 0,0	100,0	100,0; 100,0
Primeiro resultado de CD4+						
<200 células/mm^3^	33,3	20,4; 46,3	27,5	15,2; 39,7	39,2	25,8; 52,6
200-350 células/mm^3^	37,1	27,0; 47,1	33,7	23,9; 43,5	29,2	19,8; 38,7
>350 células/mm^3^	29,2	24,5; 33,9	32,2	27,4; 37,0	38,6	33,6; 43,6
Sem informação	0,0	0,0; 0,0	0,0	0,0; 0,0	100,0	100,0; 100,0
Estadiamento do HIV						
Estágio I	31,5	25,7; 37,4	30,3	24,5; 36,1	38,2	32,0; 44,3
Estágio II	24,6	16,9; 32,2	36,1	27,5; 44,6	39,3	30,7; 48,0
Estágio III	36,8	26,6; 46,9	33,3	23,4; 43,2	29,9	20,3; 39,5
Estágio IV	33,3	20,4; 46,3	27,5	15,2; 39,7	39,2	25,8; 52,6
Sem informação	0,0	0,0; 0,0	0,0	0,0; 0,0	100,0	100,0; 100,0
Esquema da TARV						
3TC+TDF+DTG	33,9	29,4; 38,3	33,6	29,2; 38,1	32,5	28,1; 36,9
3TC+TDF+EFZ	10,0	0,7; 19,3	27,5	13,7; 41,3	62,5	47,5; 77,5
Outro	14,3	-4,0; 32,6	7,1	-6,3; 20,6	78,6	57,1; 100,1
Não iniciou TARV	0,0	0,0; 0,0	0,0	0,0; 0,0	100,0	100,0; 100,0
Efeito colateral						
Não	32,0	27,5; 36,4	33,4	28,9; 37,9	34,6	30,1; 39,2
Sim	30,0	19,3; 40,7	28,6	18,0; 39,2	41,4	29,9; 53,0
Não iniciou TARV	0,0	0,0; 0,0	0,0	0,0; 0,0	100,0	100,0; 100,0
Número de esquemas						
1	32,8	28,5; 37,2	33,3	28,9; 37,6	33,9	29,5; 38,3
>1	17,4	6,4; 28,3	23,9	11,6; 36,2	58,7	44,5; 72,9
Não iniciou TARV	0,0	0,0; 0,0	0,0	0,0; 0,0	100,0	100,0; 100,0
Último resultado de CD4+						
<200 células/mm^3^	34,8	15,3; 54,2	13,0	-0,7; 26,8	52,2	31,8; 72,6
200-350 células/mm^3^	31,9	18,6; 45,2	25,5	13,1; 38	42,6	28,4; 56,7
>350 células/mm^3^	30,7	26,3; 35,1	33,7	29,3; 38,2	35,6	31,1; 40,1
Sem informação	0,0	0,0; 0,0	0,0	0,0; 0,0	100,0	100,0; 100,0
Último resultado de CV						
Suprimido	24,4	11,2; 37,5	22,0	9,3; 34,6	53,7	38,4; 68,9
Não suprimido	17,9	9,4; 26,5	14,1	6,4; 21,8	67,9	57,6; 78,3
Indetectável	34,4	29,6; 39,2	36,7	31,9; 41,6	28,9	24,3; 33,4
Sem informação	0,0	0,0; 0,0	0,0	0,0; 0,0	100,0	100,0; 100,0
Infecção oportunista						
Não	25,5	16,7; 34,3	29,8	20,5; 39,0	44,7	34,6; 54,7
Sim	31,9	27,4; 36,4	32,1	27,6; 36,6	36,0	31,4; 40,7
Sem informação	0,0	0,0; 0,0	0,0	0,0; 0,0	100,0	100,0; 100,0
Outra IST						
Não	28,7	23,2; 34,1	29,1	23,6; 34,5	42,3	36,3; 48,2
Sim	32,8	26,9; 38,7	34,4	28,4; 40,4	32,8	26,9; 38,7
Sem informação	0,0	0,0; 0,0	0,0	0,0; 0,0	100,0	100,0; 100,0
Realização de busca ativa						
Não	18,2	-4,6; 41,0	54,5	25,1; 84,0	27,3	1,0; 53,6
Sim	30,5	26,5; 34,6	30,7	26,7; 34,8	38,7	34,5; 43,0
Tempo entre diagnóstico e TARV						
<50 dias	37,8	32,0; 43,6	28,5	23,1; 33,9	33,7	28,1; 39,3
50-100 dias	28,3	20,5; 36,2	40,2	31,6; 48,7	31,5	23,4; 39,6
>100 dias	16,7	9,2; 24,1	33,3	23,9; 42,8	50,0	40,0; 60,0
Não iniciou TARV	5,3	-4,8; 15,3	0,0	0,0; 0,0	94,7	84,7; 104,8
Ausência em consulta						
Não	52,9	43,3; 62,5	27,9	19,3; 36,5	19,2	11,7; 26,8
Sim	24,5	20,3; 28,7	32,1	27,6; 36,6	43,4	38,6; 48,2

*TI - taxa de incidência; IC95% - Intervalo de Confiança de 95%; CV - carga viral; TARV - terapia antirretroviral; IST - infecção sexualmente transmissível; 3TC+TDF+EFZ - lamivudina+tenofovir+efavirenz; 3TC+TDF+DTG - lamivudina+tenofovir+dolutegravir.*

Na análise bivariada com Regressão de Poisson com variância robusta, 25 variáveis apresentaram significância estatística (p<0,25), as quais foram utilizadas para construção de modelos múltiplos, tanto para associação da má adesão quanto para perda de seguimento, conforme Tabela 2.

**Tabela 2:** Análise univariada dos fatores associados à má adesão e perda de seguimento entre adolescentes e jovens vivendo com HIV, Maringá, Paraná, Brasil, 2025

Variável	Má adesão			Perda de seguimento		
	RR	IC95%	Valor de *p*	RR	IC95%	Valor de *p*
Sexo						
Feminino	Referência		0,099	Referência		<0,001
Masculino	0,76	0,55; 1,05		0,64	0,52; 0,79	
Faixa etária						
15-19 anos	Referência		0,018	Referência		0,326
20-24 anos	0,74	0,58; 0,95		0,87	0,66; 1,14	
Estado civil						
Sem companheiro	Referência		0,779	Referência		0,013
Com companheiro	1,05	0,73; 1,49		1,34	1,06; 1,69	
Raça/cor						
Branca	Referência		0,107	Referência		0,045
Não branca	1,20	0,96; 1,52		1,23	1,00; 1,52	
Escolaridade						
≤12 anos	Referência		0,065	Referência		0,005
>12 anos	0,80	0,64; 1,01		0,72	0,57; 0,90	
Religião						
Não	Referência		0,601	Referência		0,051
Sim	1,06	0,85; 1,31		1,22	0,99; 1,49	
Orientação sexual						
Heterossexual	Referência		0,279	Referência		<0,001
Homossexual	0,89	0,63; 1,26		0,65	0,53; 0,79	
Bissexual	1,12	0,74; 1,70		0,58	0,35; 0,94	
Ocupação						
Empregado/autônomo	Referência		0,385	Referência		0,008
Estudante/do lar	1,20	0,91; 1,59		1,05	0,76; 1,45	
Desempregado	0,80	0,27; 2,36		1,55	1,17; 2,04	
Distância até serviço						
<5 km	Referência		0,154	Referência		0,007
5-10 km	0,81	0,63; 1,03		1,06	0,80; 1,41	
>10 km	1,01	0,76; 1,35		1,44	1,08; 1,92	
Pessoa em situação de rua						
Não	Referência		-	Referência		0,901
Sim	-	-		0,93	0,35; 2,51	
Pessoa privada de liberdade						
Não	Referência		0,979	Referência		0,235
Sim	0,98	0,24; 3,90		1,41	0,79; 2,51	
Uso de álcool						
Não	Referência		0,588	Referência		0,165
Sim	0,94	0,75; 1,17		0,86	0,70; 1,06	
Uso de tabaco						
Não	Referência		0,647	Referência		0,099
Sim	0,94	0,73; 1,20		1,18	0,96; 1,45	
Uso de drogas						
Não	Referência		0,845	Referência		0,197
Sim	1,02	0,80; 1,31		1,15	0,93; 1,42	
Parceria sexual						
Não	Referência		0,013	Referência		0,016
Sim	1,31	1,05; 1,62		1,27	1,04; 1,56	
Comorbidade						
Não	Referência		0,162	Referência		0,295
Sim	0,66	0,37; 1,17		0,78	0,49; 1,23	
Transtorno mental						
Não	Referência		0,608	Referência		0,284
Sim	1,07	0,80; 1,44		1,14	0,89; 1,47	
Uso de psicotrópico						
Não	Referência		0,704	Referência		0,592
Sim	1,06	0,78; 1,44		1,08	0,81; 1,42	
Entrada no serviço						
Caso novo	Referência		0,161	Referência		0,002
Caso transferido	1,19	0,93; 1,53		1,37	1,21; 1,68	
Primeiro resultado de CV						
Suprimido	Referência		0,112	Referência		<0,001
Não suprimido	0,65	0,53; 1,02		0,68	0,52; 0,89	
Indetectável	0,93	0,57; 1,52		1,06	0,77; 1,47	
Primeiro resultado de CD4+						
<200 células/mm^3^	Referência		0,633	Referência		0,280
200-350 células/mm^3^	1,05	0,66; 1,61		0,81	0,53; 1,25	
>350 células/mm^3^	1,16	0,77; 1,75		1,05	0,76; 1,46	
Estadiamento do HIV						
Estágio I	Referência		0,348	Referência		0,319
Estágio II	1,20	0,94; 1,54		1,13	0,90; 1,43	
Estágio III	0,96	0,70; 1,31		0,82	0,59; 1,14	
Estágio IV	0,91	0,60; 1,39		0,99	0,70; 1,39	
Esquema da TARV						
3TC+TDF+DTG	Referência		0,060	Referência		<0,001
3TC+TDF+EFZ	1,46	1,05; 2,03		1,78	1,47; 2,14	
Outro	0,66	0,13; 3,31		1,74	1,34; 2,26	
Efeito colateral						
Não	Referência		0,898	Referência		0,418
Sim	0,97	0,70; 1,36		1,11	0,85; 1,45	
Número de esquemas						
1	Referência		0,324	Referência		<0,001
>1	1,21	0,82; 1,78		1,58	1,28; 1,94	
Último resultado de CD4+						
<200 células/mm^3^	Referência		0,324	Referência		0,844
200-350 células/mm^3^	1,63	0,56; 4,67		0,96	0,59; 1,56	
>350 células/mm^3^	1,92	0,72; 5,08		0,90	0,60; 1,35	
Último resultado de CV						
Suprimido	Referência		0,741	Referência		<0,001
Não suprimido	0,92	0,48; 1,77		1,21	0,89; 1,64	
Indetectável	1,09	0,67; 1,78		0,70	0,51; 0,96	
Infecção oportunista						
Não	Referência		0,640	Referência		0,098
Sim	0,93	0,70; 1,23		0,82	0,65; 1,03	
Outra IST						
Não	Referência		0,919	Referência		0,055
Sim	1,01	0,81; 1,25		0,81	0,66; 1,00	
Realização de busca ativa						
Não	Referência		0,060	Referência		0,901
Sim	0,67	0,44; 1,01		1,06	0,39; 2,85	
Tempo entre diagnóstico e TARV						
<50 dias	Referência		0,002	Referência		<0,001
50-100 dias	1,63	1,06; 1,74		1,13	0,86; 1,46	
>100 dias	1,55	1,19; 2,01		1,59	1,28; 1,96	
Ausência em consulta						
Não	Referência		0,003	Referência		<0,001
Sim	1,62	1,18; 2,22		2,34	1,57; 3,48	

*RR - risco relativo; IC95% - Intervalo de Confiança de 95%; CV - carga viral; TARV - terapia antirretroviral; IST - infecção sexualmente transmissível; 3TC+TDF+EFZ - lamivudina+tenofovir+efavirenz; 3TC+TDF+DTG - lamivudina+tenofovir+dolutegravir.*

*Nota: o sinal de meia-risca indica que, para aquela subvariável, não houve possibilidade de obtenção do parâmetro para o modelo pela ausência de observações.*

No modelo multivariado final da má adesão apresentado na Tabela 3, houve evidência dos fatores negativamente associados, os quais incluíram ter parceria sexual, tempo entre diagnóstico e início da TARV variando de 50-100 dias e >100 dias, e ausência em consultas. Por outro lado, o único fator positivamente associado foi a faixa etária de 20-24 anos.

**Tabela 3:** Análise multivariada dos fatores associados à má adesão do tratamento entre adolescentes e jovens vivendo com HIV, Maringá, Paraná, Brasil, 2025

Variável		Má adesão^^*^^	
	RR	IC95%	Valor de *p*
Faixa etária			
15-19 anos		Referência	0,040
20-24 anos	0,77	0,61; 0,98	
Parceria sexual			
Não		Referência	0,027
Sim	1,26	1,02; 1,56	
Tempo entre diagnóstico e TARV			
<50 dias		Referência	0,016
50-100 dias	1,27	1,01; 1,62	
>100 dias	1,45	1,11; 1,89	
Ausência em consulta			
Não		Referência	0,007
Sim	1,52	1,12; 2,06	

RR - risco relativo; TARV - terapia antirretroviral; IC95% - Intervalo de Confiança de 95%;

* modelo múltiplo final ajustado para todas as variáveis remanescentes e para a variável “distância até serviço”.

No modelo multivariado final para perda de seguimento, apresentado na Tabela 4, os fatores negativamente associados foram uso de drogas, esquema de TARV composto por lamivudina+tenofovir+efavirenz e outro esquema, último resultado de carga viral não suprimido antes do desfecho, tempo entre diagnóstico e TARV de 50-100 dias e >100 dias, assim como ausência em consulta antes da perda de seguimento. Os fatores positivamente associados foram uso de álcool e primeiro exame de carga viral não suprimido, e último exame de carga viral indetectável.

**Tabela 4:** Análise multivariada dos fatores associados à perda de seguimento entre adolescentes e jovens vivendo com HIV, Maringá, Paraná, Brasil, 2025

Variável		Perda de seguimento^^*^^	
	RR	IC95%	Valor de *p*
Uso de álcool			
Não		Referência	0,021
Sim	0,80	0,66; 0,96	
Uso de drogas			
Não		Referência	0,026
Sim	1,25	1,02; 1,52	
Primeiro resultado de CV			
Suprimido		Referência	0,002
Não suprimido	0,73	0,54; 1,00	
Indetectável	1,20	0,86; 1,66	
Esquema da TARV			
3TC+TDF+DTG		Referência	<0,001
3TC+TDF+EFZ	1,46	1,18; 1,80	
Outro	1,63	1,27; 2,09	
Último resultado de CV			
Suprimido		Referência	<0,001
Não suprimido	1,12	0,83; 1,53	
Indetectável	0,66	0,49; 0,91	
Tempo entre diagnóstico e TARV			
<50 dias		Referência	0,002
50-100 dias	1,15	0,90; 1,47	
>100 dias	1,46	1,18; 1,79	
Ausência em consulta			
Não		Referência	0,002
Sim	1,84	1,24; 2,71	

RR - risco relativo; IC95% - Intervalo de Confiança de 95%; CV - carga viral; TARV - terapia antirretroviral; 3TC+TDF+EFZ - lamivudina+tenofovir+efavirenz; 3TC+TDF+DTG - lamivudina+tenofovir+dolutegravir;

* modelo múltiplo final ajustado para todas as variáveis remanescentes e para a variável “distância até serviço”.

## DISCUSSÃO

O estudo identificou que a taxa de insucesso do tratamento entre adolescentes e jovens vivendo com HIV, estabelecida pela má adesão e perda de seguimento, foi de 78,4% e 68,3%, respectivamente. Para enfrentar esses desafios, são necessários esforços para mitigar e compreender os fatores de risco e, assim, melhorar a adesão ao tratamento.

Como observado, mais de dois terços dos adolescentes e jovens em tratamento não aderiram totalmente à TARV em algum momento do seguimento. Esse achado ultrapassa o limite de 5% estabelecido pela Organização Mundial da Saúde (OMS)^(14)^. Embora os medicamentos sejam gratuitos, usuários financeiramente desfavorecidos enfrentam dificuldades no acesso aos cuidados, devido a custos de transporte, e perda de renda por afastamento do trabalho e outros desafios logísticos^(15)^.

Os adolescentes entre 15 e 19 anos estão cada vez mais ativos sexualmente. Isto implica apreensões sobre a revelação inadvertida ao parceiro sexual se forem vistos nos serviços de HIV ou tomando o medicamento antirretroviral^(15)^. Este contexto faz com que a parceria sexual se torne um fator para má adesão, em decorrência do medo da revelação do diagnóstico, estigma e preconceito, trazendo, consequentemente, reflexos negativos sobre adesão ao tratamento.

O uso de drogas ilícitas tem sido consistentemente identificado como fator para perda de seguimento, embora o tipo específico de substância usada por esses adolescentes e jovens nem sempre seja relatado. Estudo com usuários brasileiros do Hornet (aplicativo e rede social para homens *gays*) indica que, entre minorias sexuais, a associação entre uso de cannabis e adesão à TARV ainda não foi evidenciada, enquanto o uso de cocaína, heroína e metanfetaminas está negativamente associado à adesão^(16)^. Dessa forma, a triagem para uso de substâncias durante o tratamento do HIV tem se mostrado eficaz para identificar transtornos e encaminhar para tratamento adequado^(16)^.

Em relação ao esquema de TARV, observou-se que combinações baseadas em efavirenz e outros antirretrovirais, exceto o dolutegravir, representaram maior propensão à falha na carga viral. Estudo destaca os benefícios do regime com efavirenz na adesão e supressão virológica^(17)^. Cabe ressaltar que a coorte iniciou em 2017, quando a OMS aprovou a transição do esquema com efavirenz para o dolutegravir, indicando a necessidade de novos estudos que explorem essa associação.

Quando não há uma boa adesão ao tratamento, a carga viral tende a permanecer elevada. Nessa perspectiva, o último resultado de carga viral não suprimido se associa negativamente ao desfecho da perda de seguimento. Esse preditor pode estar relacionado à baixa renda do paciente, que compromete seus cuidados de saúde e exige o deslocamento para obtenção da medicação, resultando em doses perdidas e maior risco de falha virológica^(18)^. Estudo realizado no Zimbábue identificou que a baixa renda estava associada à alta carga viral (≥1.000 cópias/mL) em pacientes em TARV^(19)^.

Já o tempo entre diagnóstico e TARV de 50-100 dias e >100 dias foi um fator tanto para má adesão quanto para perda de seguimento. Esse resultado chama atenção, uma vez que a OMS sugere o início da TARV no mesmo dia do diagnóstico^(20)^. Porém, apresentou que as taxas de perda de seguimento têm sido mais elevadas em países em desenvolvimento^(21)^. Além disso, a Sociedade Internacional de Antirretrovirais também recomenda especificamente o início da TARV dentro de 14 dias para PVHIV que têm infecções oportunistas^(22)^. Estudo realizado em Pattaya e Bangkok identificou que o início da TARV dentro de cinco dias da infecção pelo HIV aumentou a probabilidade de ter DNA do HIV indetectável nas células CD4+^(23)^.

Dessa forma, é imprescindível o diagnóstico precoce do HIV e, tão logo, o início do tratamento como medida benéfica para a saúde do indivíduo e para a sociedade, uma vez que contribuirá para prevenção da transmissão. Os testes de rotina para infecções sexualmente transmissíveis têm um papel essencial na identificação e tratamento de muitas infecções que não poderiam ser detectadas e tratadas em indivíduos assintomáticos^(24)^.

Os pacientes com um comportamento faltoso tendem à má adesão e perda de seguimento. Por exemplo, estudo na Etiópia identificou que os adolescentes e jovens que perderam suas consultas provavelmente perderiam suas doses e aumentariam as chances de falha virológica^(25)^.

No presente estudo, ter entre 20 e 24 anos foi fator protetor para má adesão, o que diverge do observado em outras pesquisas^(15,26)^. Com o avanço da idade, a percepção do estigma relacionado ao HIV pode reduzir a disposição em revelar o *status* sorológico e em utilizar os serviços de saúde. Além disso, o cansaço psicológico, físico, cognitivo e até sexual pode afetar a adesão ao tratamento^(15)^.

Os resultados do estudo atual divergem da literatura também no que concerne à associação entre o uso de álcool e perda de seguimento. A literatura evidencia que aqueles com histórico de uso de álcool são mais propensos a faltar às consultas clínicas e não aderir à TARV. Além disso, o uso de álcool não está apenas associado ao insucesso do tratamento, mas também é considerado um importante fator para morbidade e mortalidade na América Latina^(16,27)^. A relação entre o uso de álcool e perda de seguimento no cenário brasileiro merece uma exploração mais aprofundada, uma vez que, neste estudo, o uso do álcool foi positivamente associado à perda de seguimento.

O primeiro exame de carga viral não suprimido foi um fator de proteção para perda de seguimento, fazendo com que o adolescente e/ou jovem veja a necessidade de continuar o tratamento para alcançar a carga viral indetectável^(28)^. Já quando o resultado do último exame de carga viral se torna indetectável, os jovens que estão em perda de seguimento veem a necessidade de manter os cuidados com HIV para ter qualidade de vida.

Assim, profissionais de saúde devem avançar nas interações com as PVHIVs, a fim de aumentar a conscientização dos conceitos do I=I, ou seja, indetectável é igual à intransmissível, sendo uma expressão científica em que as PVHIVs em tratamento não irão transmitir o vírus. Assim, uma melhor compreensão deste conceito tem o potencial de desafiar o estigma e, por sua vez, apoiar uma maior adesão ao tratamento do HIV^(29)^.

### Limitações do estudo

Por se tratar de estudo de coorte ambispectiva, no qual as informações dos pacientes foram extraídas dos prontuários de suas consultas de rotina e retiradas dos medicamentos, sabe-se que a integralidade dos dados depende da robustez da coleta de dados realizada pela equipe do ambulatório e, portanto, sujeita a falhas no registro de informações. Ainda assim, os resultados encontrados poderão subsidiar a elaboração de estratégias voltadas para aprimoramento do atendimento a adolescentes e jovens vivendo com HIV.

Além disso, a amostra incluiu todos os adolescentes e jovens vivendo com HIV atendidos no ambulatório que presta atendimento a 30 municípios, o que reduz possíveis vieses de seleção e confere validade interna considerando a qualidade das evidências. Em relação à validade externa, o estudo permite a generalização dos resultados em contextos similares ao do serviço em que foi desenvolvido esta pesquisa. No entanto, sugere-se a realização de pesquisas multicêntricas no território brasileiro com finalidade de obtenção de um panorama nacional.

### Contribuições para as áreas da enfermagem, saúde ou políticas públicas

Os resultados deste estudo apresentam potencial contribuição para a saúde pública, ao possibilitar a identificação de situações de vulnerabilidade e dos determinantes associados à má adesão e perda de seguimento no tratamento do HIV em adolescentes e jovens. Assim, podem subsidiar a reformulação de políticas de saúde voltadas à melhoria dos serviços e das estratégias de cuidado, contribuindo para o alcance da meta de eliminação da aids até 2030.

## CONCLUSÕES

A incidência de má adesão e perda de seguimento entre adolescentes e jovens vivendo com HIV é elevada, e está associada, principalmente, a fatores socioeconômicos e clínico-epidemiológicos. Esses resultados reiteram os diversos determinantes imbricados ao adoecimento e tratamento dessa condição, e apontam a necessidade de estratégias que visem à adesão e retenção aos cuidados de HIV entre os grupos mais vulneráveis a esses desfechos, como adolescentes e jovens.

## Data Availability

Os dados de pesquisa só estão disponíveis mediante solicitação.
